# *Premna odorata*: Seasonal Metabolic Variation in the Essential Oil Composition of Its Leaf and Verification of Its Anti-Ageing Potential via In Vitro Assays and Molecular Modelling

**DOI:** 10.3390/biom10060879

**Published:** 2020-06-08

**Authors:** Ahmed E. Altyar, Mohamed L. Ashour, Fadia S. Youssef

**Affiliations:** 1 Department of Pharmacy Practice, Faculty of Pharmacy, King Abdulaziz University, P.O. Box 80260, Jeddah 21589, Saudi Arabia; aealtyar@kau.edu.sa; 2 Department of Pharmacognosy, Faculty of Pharmacy, Ain-Shams University, Cairo 11566, Egypt; fadiayoussef@pharma.asu.edu.eg

**Keywords:** anti-ageing, essential oil, GC/MS, molecular modelling, *Premna odorata*, Verbenaceae

## Abstract

The metabolic variation in the essential oil composition of *Premna odorata* leaves obtained from different seasons was quantitatively and qualitatively determined employing GC/MS (Gas Chromatography coupled with Mass Spectrometry) and GC/FID (Gas chromatography equipped with flame ionization detector) techniques. It displayed the existence of 97 constituents accounting for 94.19%, 92.27%, 91.95% and 92.63% for POS (spring), POM (summer), POA (autumn) and POW (winter) whole essential oils. *β*-Caryophyllene constituting the main metabolite in the oil in the different seasons. To better visualize the differences between them, GC data were exposed to chemometric analysis. A PCA (principal component analysis) score plot revealed the closeness of POS and POW. Molecular modelling on collagenase, elastase and hyaluronidase enzymes active centres shows that different compounds existing in the essential oil of *Premna odorata* leaves shows binding to the active sites with variable degrees that suggested its anti-ageing potential. Palmitic acid displayed the highest fitting for both the collagenase and elastase active centres in both pH-based and rule-based ionization methods with ∆G equals −78.27 and −44.77 kcal/mol, respectively; meanwhile, heptacosane showed the highest fitting score in the hyaluronidase centre with ∆G = −43.78 kcal/mol. In vitro assays consolidates the obtained modelling studies in which essential oil shows considerable anti-elastase and anti-hyaluronidase potential as evidenced by their IC_50_ values being 49.3 and 37.7 μg/mL, respectively; meanwhile, the essential oil of *Premna odorata* leaves displayed mild anti-collagenase potential. Thus, it can be concluded that *Premna odorata* could serve as a promising anti-ageing naturally occurring drug that could be effectively incorporated by pharmaceutical industries in cosmetics combating ageing and skin wrinkling.

## 1. Introduction

Ageing is primarily caused by the pronounced disturbance in the balance between the regenerative and the degenerative potential of the skin causing wrinkling and thinning of its epidermis. This is subsequently accompanied by the appearance of crevices, furrows, lines and creases in the lines of facial expression in particular. Basically, ageing appears due to the extensive morphological alteration in the dermal layer triggered by repeated exposure to UV radiations and free radicles as well. This alteration is accompanied by extensive loss in collagen as well as elastin fibres with reduced circulatory perfusion [1].

Many efficient anti-ageing products are derived from natural sources, they succeeded in restoring the disturbed balance between regenerative/degenerative power of the skin in addition to potentiating the production of elastin and collagen as ginseng meanwhile others cause transient moisturizing effects [1].

Genus *Premna* belongs to family Verbenaceae, comprising nearly 200 species. It grows natively in the Pacific islands, Africa, Australia and Asia, particularly in the tropical as well as the subtropical areas. Traditionally, many members belonging to genus *Premna* are used to cure gastrointestinal disorders, such as diarrhoea and liver ailments. In addition, recent studies showed they possess a great therapeutic potential represented by their antimicrobial, antioxidant, cytotoxic, anti-inflammatory and hepatoprotective activity. These activities may be relied upon their richness with several classes of compounds, such as flavonoids, lignans, isoflavones, triterpenoids, diterpenoids, sesquiterpenoids, as well as iridoids [2].

*Premna odorata* constitutes mainly shrubs and trees and was traditionally employed as irrigation for vaginal disorders and in the alleviation of tuberculosis as well. In spite of the richness of genus *Premna* with many secondary metabolites to which many of its biological activities were attributed. It was noticed that few were traced in literature regarding the biological activities and the phytoconstituents of *Premna odorata.* Few iridoid glycosides and flavonoids were isolated from its leaf in addition to the investigation of its essential oil for its anti-tuberculosis activity [3].

This study aimed to comprehensively determine the metabolic variation in the essential oil composition of *Premna odorata* leaves between different seasons using gas chromatography data coupled with a chemometric study using principal component analysis (PCA). Meanwhile in silico and in vitro experiments were used to assess the interaction between metabolites and enzymes. The major constituents will be subjected to in silico virtual screening within the active sites of collagenase, elastase and hyaluronidase enzymes to examine its anti-ageing potential and to find out their exact mode of action and their probable behaviour with the binding site if any. Furthermore, the essential oil will be further examined for its anti-ageing potential via inhibiting these enzymes using various in vitro assays with the aim of finding new resources that can be incorporated in pharmaceutical cosmetics for combating ageing.

## 2. Materials and Methods

### 2.1. Plant Material

The leaves of *Premna odorata* Blanco (Family Verbenaceae) were collected from Zoo botanical garden during the four seasons spring, summer, autumn and winter 2018. They were kindly authenticated and confirmed morphologically by Mrs. Terease Labib, Consultant of Plant Taxonomy at Ministry of Agriculture, El-Orman Botanical Garden and National Gene Bank, Giza, Egypt. Voucher materials representing leaves from spring, summer, autumn and winter were kept as a herbarium in Pharmacognosy Department, Faculty of Pharmacy, Ain Shams University under the codes (PHG-P-PO-426 (POS); PHG-P-PO-427(POM); PHG-P-PO-428 (POA) and PHG-P-PO-429 (POW)), respectively.

### 2.2. Preparation of Essential Oil Samples

The fresh leaves of *Premna odorata* obtained from spring, summer, autumn and winter was air dried in the lab (at temperature not exceeding 35 °C). The essential oil samples were prepared via the hydrodistillation of the air-dried leaves of *Premna odorata* obtained from spring, summer, autumn and winter using Clevenger-type apparatus for 6 h. Essential oils POS, POM, POA and POW were dehydrated using anhydrous Na_2_SO_4_ for spring, summer, autumn and winter samples to yield 0.031, 0.025, 0.018 and 0.022% *v*/*w* of dry weight, respectively. The different oil samples were analyzed directly by GC/FID and GC/MS and then kept in dark-coloured stoppered glasses at −30 °C for further biological analyses [4].

### 2.3. GC/FID and GC/MS Analyses

Shimadzu GC-17A gas chromatograph (Shimadzu Corporation, Kyoto, Japan) with a DB-5 fused-bonded cap column (Phenomenex; 29 m × 0.25 mm i.d., film thickness 0.25 µm; Torrance, CA, USA) and a FID detector was used for the quantitative analysis of the samples. Shimadzu GC-2010 plus a gas chromatograph (Shimadzu Corporation, Kyoto, Japan) were employed for GC analyses and equipped with Rtx-5MS (Restek, Bellefonte, PA, USA) and a quadrupole mass spectrometer for identification of the volatile oil components. Instrumental settings were adjusted according to what was previously reported [5]. Three independent runs were used to calculate AUP (areas under the peaks) using Class GC 10^®^ software (Shimadzu Corporation, Kyoto, Japan) in which the total area is considered 100%. Meanwhile, for qualitative interpretation of the chromatograms, GC solution^®^ software ver. 2.4 (Shimadzu Corporation, Kyoto, Japan) was used. For comparing and identifying the constituents of the different essential oil samples, Wiley Registry of Mass Spectral Data 8th edition, NIST MassSpectral Library (December 2011), and previously reported techniques were employed [6].

### 2.4. Chemometric Analysis

To better visualize the differences between the compositions of essential oils obtained from the leaves of *Premna odorata* collected at different seasons, the GC data were exposed to unsupervised pattern recognition chemometric analysis represented by principal component analysis (PCA). This was done using Unscrambler^®^ 9.7 (CAMO SA, Oslo, Norway) as previously described [7,8]. Additionally, a heat map was also done using GC data that was processed using Hierarchical Clustering Explorer 3.5 software (Human computer interaction laboratory, University of Maryland, College Park, MD, USA) [7,8].

### 2.5. In Silico Virtual Studies

In silico virtual screening was performed inside the active centres of three crucial enzymes implicated in the occurrence and progression of ageing process *viz*. collagenase (PDB ID: 2D1N; 2.37 Å), elastase (PDB ID: 1BRU; 2.30 Å) and hyaluronidase (PDB ID: 1FCV; 2.65 Å) using Discovery Studio 4.5 (Accelrys Inc., San Diego, CA, USA) applying the C-Docker protocol. The X-ray crystal structures of the utilized enzymes were downloaded from a protein data bank (www.pdb.org) in PDB format. Each enzyme structure was prepared using the default protocol for protein preparation of Discovery Studio 4.5 (Accelrys Inc., San Diego, CA, USA) where water molecules were removed, hydrogen atoms were added followed by the cleanliness of the protein structure from any undesirable interactions. CHARMm was chosen as the forcefield meanwhile MMFF94 was used as a method for partial charge calculation followed by minimization of the added hydrogen in about 2000 steps. The binding centre was determined based upon the reported data near the enzyme catalytic domain. All the compounds 2D structures were drawn using ChemDraw 15.0 and saved as PDB files. The structures were prepared using the default protocol for ligand preparation of Discovery Studio 4.5 (Accelrys Inc., San Diego, CA, USA) using both pH-based and rule-based ionization methods. Then the prepared compounds were docked within the active sites of the energy-minimized protein applying C-Docker protocol leaving its parameters as its default parameters. CHARMm force field was assigned and the binding energies were calculated using a distance dependent dielectric implicit solvation model for the selected docking poses. However, the free binding energies were calculated in kcal/mol using the following equation: [9,10].

Δ*G*_binding_ = E_complex_ − (E_protein_ + E _ligand_) where;

Δ*G*_binding_: The ligand–protein interaction binding energy,

E_complex_: The potential energy for the complex of protein bound with the ligand,

E_protein:_ The potential energy of protein alone and

E_ligand_: The potential energy for the ligand alone

### 2.6. Assessment of the Anti-Ageing Potential of Premna Odorata Leaves Essential Oil

#### 2.6.1. Anti-Collagenase Evaluation

Assessment of the collagenase inhibitory activity was measured spectrophotometrically as previously described [11]. Briefly, collagenase from *Clostridium histolyticum* (ChC-EC.3.4.23.3) was solubilized in 50 mM Tricine buffer (pH 7.5 with 400 m MNaCl and 10 mM CaCl_2_) to form 0.8 units/mL as an initial concentration in accordance to data given with 0.8 units/mL. The prepared enzyme in the buffer was added to the essential oil samples in a range of concentrations (1000–7.81 µg/mL) for 15 min, then 2 mM of the synthetic substrate *N*-[3-(2-furyl) acryloyl]-Leu-Gly-Pro-Ala (FALGPA) in Tricine buffer was added to the samples. Absorbance was continuously measured for 20 min after the immediate addition of the substrate at 335 nm by a Microplate reader. Negative control was prepared using water; meanwhile, EGCG (epigallocatechin gallate) was employed as a positive control. The IC_50_ is the concentration of sample used to inhibit 50% of collagenase under the assay conditions.

The percentage of collagenase inhibition (%) $=\left(1-\frac{S}{C}\right)\times 100$;

***S***: is the corrected absorbance of the samples; ***C***: is the corrected absorbance of the control.

#### 2.6.2. Anti-Elastase Evaluation

Estimation of elastase inhibitory activity was determined spectrophotometrically as previously reported [12]. Pancreatic elastase was solubilized in sterile water to prepare a stock solution of 3.33 mg/mL. N-Succinyl-Ala-Ala-Ala-*p*-nitroanilide was prepared by its dissolution in mM Tris-HCL buffer with pH equals 8.0 to reach 1.6 mM. The substrate was added to the samples in the range of (1000–7.81 µg/mL) after being incubated with the enzyme for 15 min. Absorbance was continuously measured for 20 min after the immediate addition of the substrate at 381–402 nm by a Microplate reader. Negative control was prepared using water; meanwhile, EGCG (epigallocatechin gallate) was employed as a positive control. The IC_50_ is the concentration of sample used to inhibit 50% of elastase under the assay conditions.

The percentage of elastase inhibition (%) $=\left(1-\frac{S}{C}\right)\times 100$;

***S***: is the corrected absorbance of the samples; ***C***: is the corrected absorbance of the control.

#### 2.6.3. Anti-Hyaluronidase Evaluation

Determination of hyaluronidase inhibitory potential was determined spectrophotometrically as previously reported using Type-1-S bovine hyaluronidase (Sigma Aldrich, St. Louis, MO, USA) [13]. Absorbance was measured at 585 nm by a Microplate reader. Negative control was prepared using water; meanwhile, EGCG (epigallocatechin gallate) was employed as a positive control. The IC_50_ is the concentration of sample used to inhibit 50% of hyaluronidase under the assay conditions.

The percentage of elastase inhibition (%) $=\left(1-\frac{S}{C}\right)\times 100$;

***S***: is the corrected absorbance of the samples; ***C***: is the corrected absorbance of the control.

### 2.7. Statistical Analysis

Data is represented in the form of mean ± SD meanwhile the IC_50_ values were computed employing Microsoft Excel 2007 package in which the level of significance is *p* < 0.05.

## 3. Results and Discussion

### 3.1. GC/FID and GC/MS Analyses

**Figure 1:**
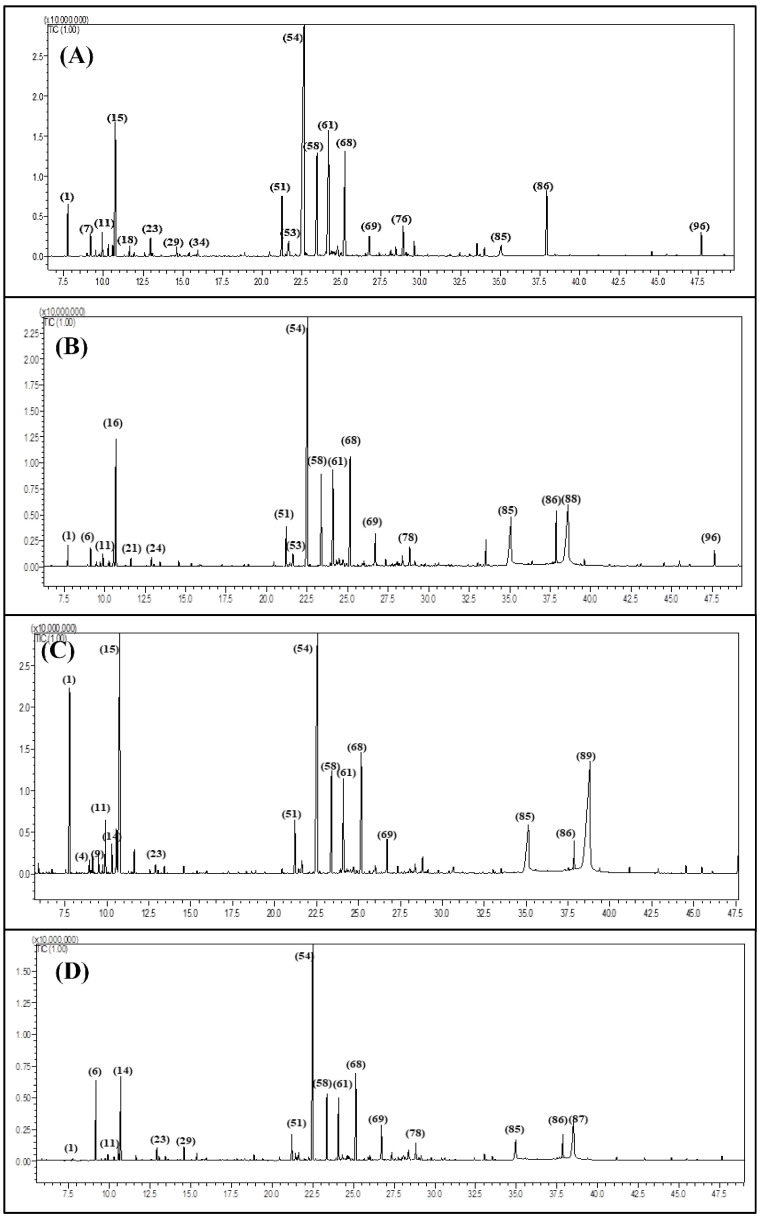
Gas Chromatography (GC) chromatograms of POS (**A**), POM (**B**), POA (**C**) and POW (**D**) using Rtx-5MS column.

**Figure 2:**
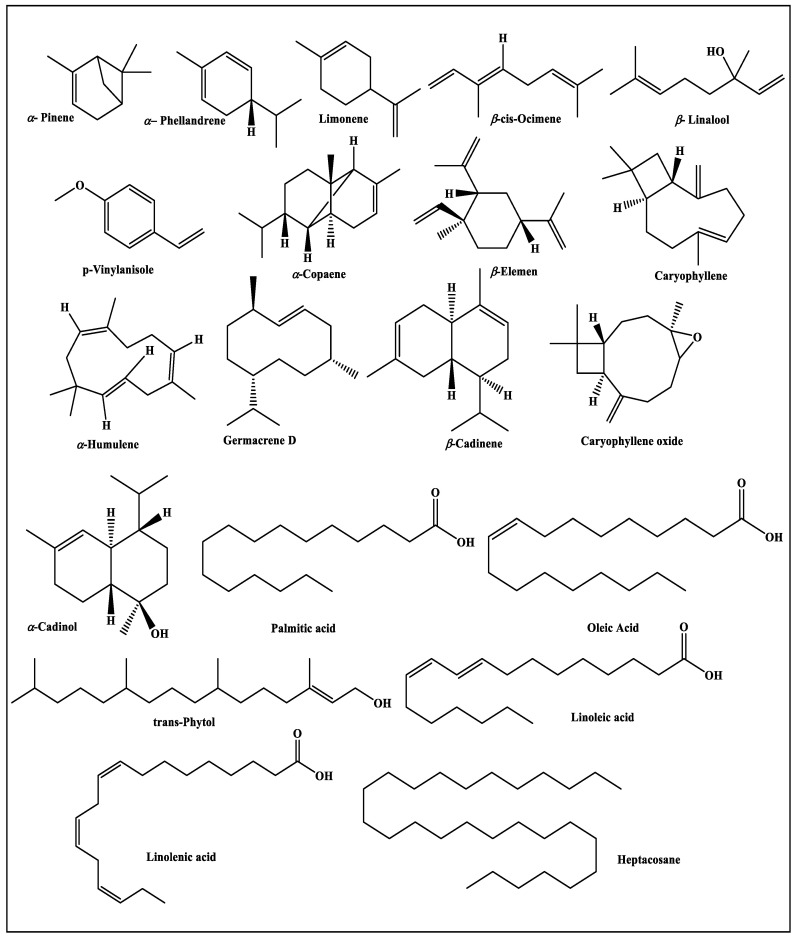
Major constituents prevailing in essential oils of POS, POM, POA and POW.

The metabolic variation in the essential oil composition of *Premna odorata* leaves obtained from different seasons was quantitatively and qualitatively determined employing GC/MS as well as GC/FID techniques. The essential oils POS, POM, POA and POW representing spring, summer, autumn and winter samples yielded 0.031, 0.025, 0.018 and 0.022% *v*/*w* of dry weight, respectively. All of the oils are yellow in colour with agreeable aromatic odour. Gas chromatographic analysis of POS, POM, POA and POW displayed the existence of 97 constituents accounting for 94.19%, 92.27%, 91.95% and 92.63% for POS, POM, POA and POW whole essential oils, respectively (Figure 1 and Figure 2). Seventy-eight metabolites were determined from POS with caryophyllene (35.37%) constituting the main metabolite followed by germacrene D (10.21%), limonene (8.34%), *β*-cadinene (7.82%), *α*-humulene (7.29%), *trans*-phytol (3.91%), *α-*copaene (3.04%), *α-*pinene (1.95%), caryophyllene oxide (1.32%), heptacosane (1.15%) and palmitic acid (1.15%). Meanwhile, fifty-one compounds were identified in POM oil with also caryophyllene (26.39%) represents the major constituent followed by linoleic acid (11.92%), palmitic acid (7.99%), *β-cis* ocimene (7.4%), *β*-cadinene (7.12%), germacrene D (6.35%), *α*-humulene (5.57%), *trans*-phytol (3.05%), caryophyllene oxide (2.31%) and *α-*copaene (2.21%). Regarding POA, sixty-four volatile constituents were detected with caryophyllene as the main metabolite representing 18.84%, followed by linolenic acid (15.76%), limonene (14%), *α-*pinene (6.85%), palmitic acid (5.75%), *β*-cadinene (5.5%), *α*-humulene (4.02%), *o*-cymene (2.16%), *α-*copaene (2.08%), *α*-phellandrene (1.57%) and caryophyllene oxide (1.46%). Furthermore, fifty-seven compounds were found in POW essential oil in which caryophyllene is the major compound accounting for 26.97% followed by oleic acid (8.9%), *β*-cadinene (7.71%), limonene (6.95%), 3-octenol (6.52%), *α*-humulene (5.75%), germacrene D (5.65%), caryophyllene oxide (3.75%), palmitic acid (3.06%), *α-*copaene (2.21%), *trans*-phytol (2%), *β*-linalool (1.21%), and *α-*cadinol (1.13%) and *p*-vinylanisole (1.04%). From the data illustrated in Table 1, it is clearly obvious that sesquiterpenes represent the prevailing volatile constituents existing in all seasons representing 67.29%, 49.08%, 36.14% and 50.6% in POS, POM, POA and POW, respectively. Meanwhile, monoterpenes represent the second class of prevailing terpenes in the essential oil of the leaves collected in spring and autumn with 13.26% and 26.72%, respectively. On the contrary, other constituents represented by fatty acids are the second class of prevailing terpenes in the summer and winter oils with 26.79% and 22.55%, respectively. Thus, it is noteworthy to highlight that differences in climatic condition greatly influence the quantity and quality of volatile constituents prevailing in the essential oil sample.

### 3.2. Chemometric Analysis

**Figure 3:**
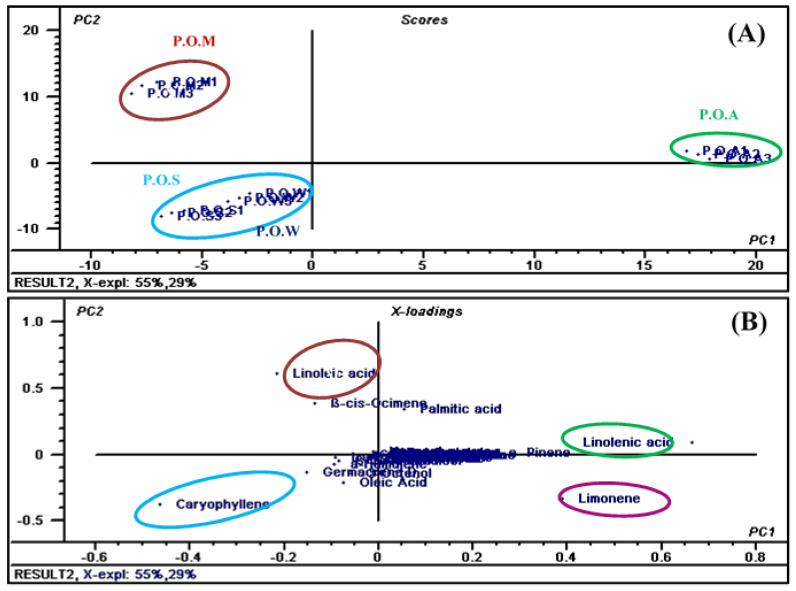
Score plot (**A**) and loading plot (**B**) of GC data obtained from POS, POW, POM and POA essential oil analyses using PCA.

**Figure 4:**
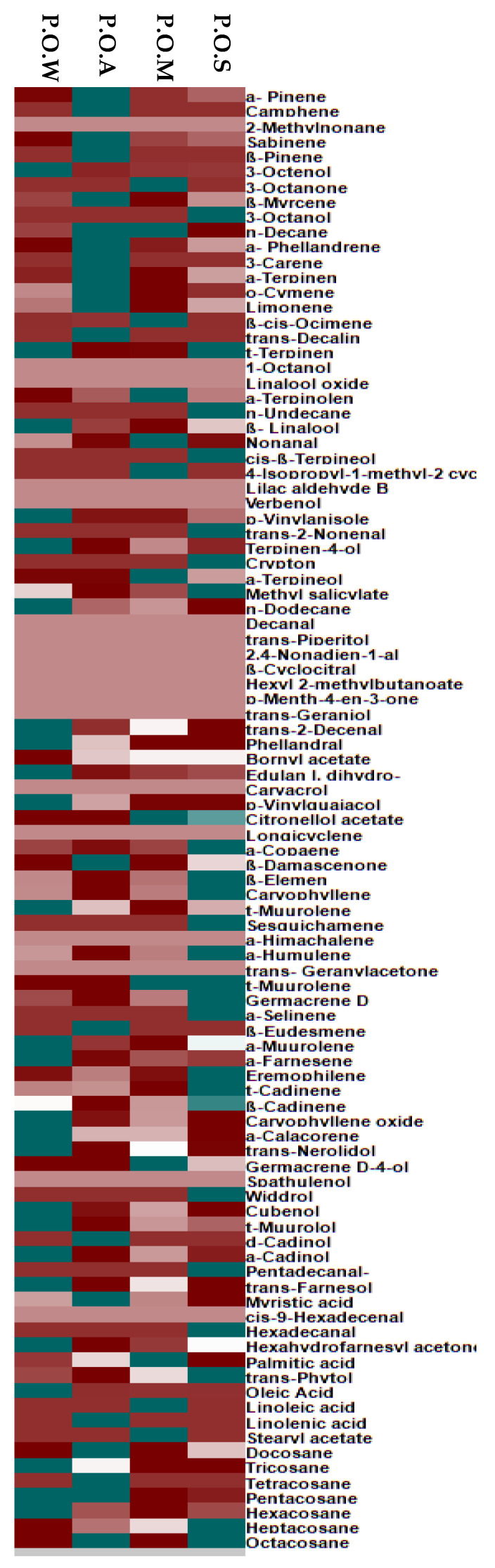
A heatmap comparison relied upon the abundance of individual components of P.O.S, P.O.M, P.O.A. and P.O.W; dark-red colour indicated non-detectable levels of the identified component.

There are multiple differences between the qualitative and quantitative compositions of essential oils obtained from the leaves of *Premna odorata* collected at different seasons that cannot be easily detected by the naked eye. Thus, to better visualize the differences between them, the data resulted from GC analyses were exposed to chemometric analysis using unsupervised pattern recognition chemometric analysis represented by Principal component analysis (PCA). The score plots obtained from PCA were illustrated in Figure 3A in which PCA considerably differentiated the examined essential oil samples into four clusters significantly discriminated along the first component (PC1) and second component (PC2) that account for 55% and 29%, respectively representing 84% of the total variances existing along the obtained data. PCA score plot revealed the closeness between the essential oil collected of *Premna odorata* leaves collected in spring and that in winter evidenced by the presence of their clusters grouped together in the lower quadrant at the left hand side of the plot. Meanwhile, both PC1 and PC2 effectively discriminated between POS and POW, which were located in the lower quadrant at the left hand side that show negative values of both PC1 and PC2 and POA that is placed in the upper quadrant at the right hand side of the plot and displayed positive values for both components. However, PC2 could be perfectly differentiated between the clusters of POS and POW that show negative values of PC2 and that of POM that is located upper quadrant at the left hand side of the plot that shows the positive value of PC2. Furthermore, PC1 successfully differentiated between POM (negative value of PC1) and POA (positive value of PC1). The loading plot represented in Figure 3B showed that the main discriminatory markers are caryophyllene, limonene, linoleic and linolenic acid. In addition, a heatmap was constructed from GC data, the amounts decrease gradually by moving from dark blue (highest amounts), light blue, white, light red till they become undetectable as represented by small dark red squares (pixels) and this promptly gives immediate visualization of information for easier understanding of the complexity of data (Figure 4).

### 3.3. In Silico Virtual Studies

For determination of the anti-ageing potential of *Premna odorata* leaves essential oil, molecular modelling for its major identified components was performed inside the active pockets of three crucial enzymes implicated in the occurrence and progression of the ageing process, namely collagenase, elastase and hyaluronidase enzymes (Table 2). Collagen represents one of the main building units of the skin and is the major constituents of the nails, hairs and connective tissue as well. It plays a pivotal role in maintaining the elasticity, flexibility and strength of the dermal tissue as well. Meanwhile, it is degraded by collagenase enzymes, so prohibition of the collagenase enzyme perfectly retards pre-collagen fibres formation with subsequent inhibition to the wrinkling process [14,15]. In addition, elastin constitutes a protein that exists in connective tissue and carries the responsibility of keeping the skin elastic that is degraded by intracellular elastase enzymes that upon elevation, either by age or continual exposure to UV-radiation, leads to ageing. In addition, hyaluronic acid performs a crucial role in keeping the elasticity, moisture and structure of the skin via facilitating the transportation of nutrients as well as waste products. It is also incorporated in fast tissue division, renewal and repair [14,15]. Loss of dermal strength and elasticity due to destruction of collagen, elastin as well as hyaluronic acid results in the appearance of wrinkle that is a characteristic feature of ageing, thus inhibition of collagenase, elastase and hyaluronidase as well as the promotion of collagen, elastin as well as hyaluronic acid production have been recently adopted as therapeutic approaches in combating ageing. Naturally occurring substances derived from the plant kingdom have been widely employed to prohibit these enzymes and stimulate collagen, elastin and hyaluronic acid formation [14,15].

**Figure 5:**
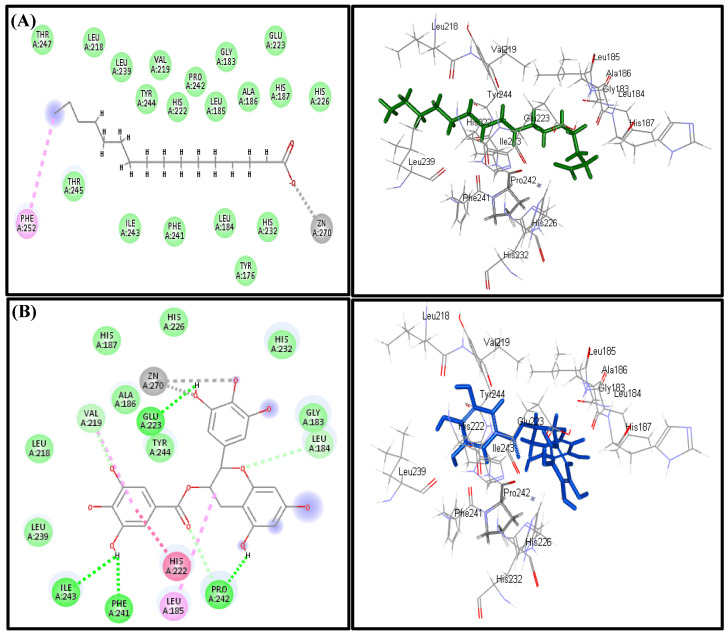
2D and 3D binding modes of Palmitic acid (**A**) and epigallocatechin gallate (EGCG) (**B**) in the active sites of the collagenase enzyme.

Regarding the collagenase enzyme, some of the identified constituents revealed tight fitting with the active sites as evidenced by the free binding energy (∆G) in which palmitic acid displayed the highest fitting in both pH-based and rule-based ionization methods with ∆G equals −78.27 kcal/mol showing a superior binding score comparable to the positive standard EGCG (∆G = −59.27 kcal/mol). Palmitic acid is followed by heptacosane (−63.64 kcal/mol), linoleic acid (−59.31 kcal/mol), oleic acid (−57.81 kcal/mol), linolenic acid (−29.85 kcal/mol), *trans*-phytol (−28.44 kcal/mol), *p-*vinylanisole (−20.06 kcal/mol), caryophyllene oxide (−7.19 kcal/mol) and *β*-linalool (−3.84 kcal/mol), meanwhile others, which show positive values of ∆G, revealed unfavorable interactions (Table 2). The tight binding of palmitic acid at the active sites can be interpreted in virtue of formation of a π-bond with Phe 252, a metal acceptor bond with the Zn 270 present at the active site in addition to the formation of multiple hydrophobic interactions and its size that fits the active site. Meanwhile, EGCG forms four hydrogen bonds with Ile 243, Phe 241, Pro 242 and Glu 223, π-π interaction with His 222, π-alkyl interaction with Leu 185, Val 219, Van der Waals interaction with Leu 184 and Pro 242 and a metal acceptor bond with the Zn 270; thus, it is clear that the latter is crucial for the interaction (Figure 5).

**Figure 6:**
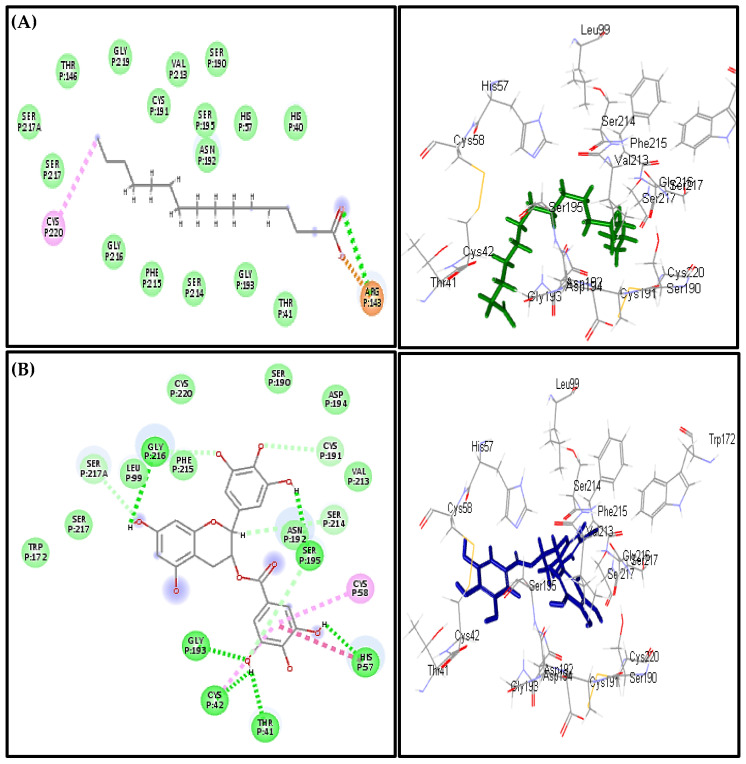
2D and 3D binding modes of Palmitic acid (**A**) and EGCG (**B**) in the active sites of the elastase enzyme.

Concerning the elastase enzyme, palmitic acid also exhibited the highest fitting score in both pH-based and rule-based ionization methods with ∆G equals −44.77 kcal/mol approaching that of EGCG (∆G = −49.35 kcal/mol). This is followed by heptacosane (−40.48 kcal/mol), linoleic acid (−31.51 kcal/mol), oleic acid (−31.50 kcal/mol), *trans*-phytol (−16.62 kcal/mol), *p*-vinylanisole (−15.36 kcal/mol) and linolenic acid (−1.43 kcal/mol) (Table 2). The strict binding of palmitic acid to the active centres can be attributed to the formation of H-bond between its carbonyl group and Arg 143, a salt bridge between the carboxylic acid group and Arg 143 in addition to the formation of multiple hydrophobic interactions and its size that fits the active site; however, EGCG forms six H-bonds with Gly 193, Cys 42, Thr 41, His 57, Ser 195 and Gly 216, Van der Waals interaction with Cys 191, Ser 195 and Ser 217, π-π interaction with His 57, π-alkyl interaction Cys 58 (Figure 6).

**Figure 7:**
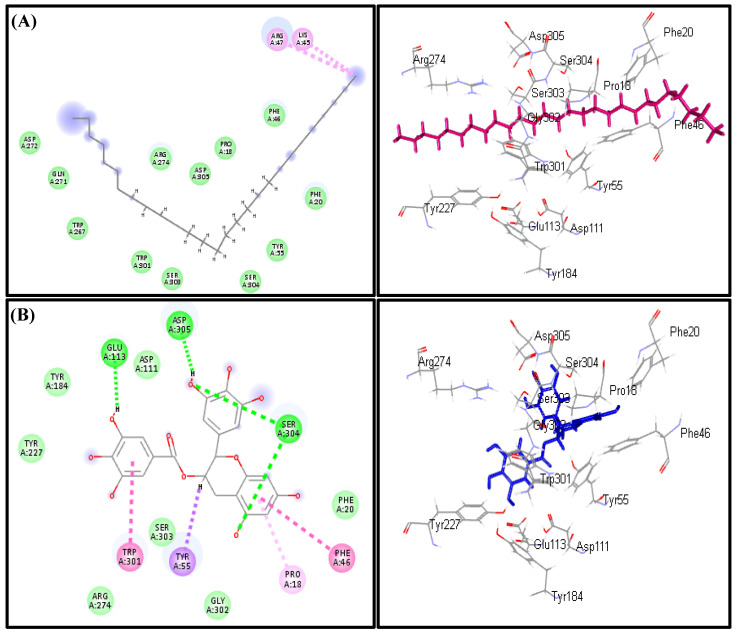
2D and 3D binding modes of Palmitic acid (**A**) and EGCG (**B**) in the active sites of the hyaluronidase enzyme.

Regarding hyaluronidase, heptacosane also displayed the highest fitting score in both pH-based and rule-based ionization methods with ∆G equals −43.78 kcal/mol approaching that of EGCG (∆G = −43.70 kcal/mol). This is followed by palmitic acid (−40.85 kcal/mol), oleic acid (−31.59 kcal/mol), linoleic acid (−30.48 kcal/mol), *p*-vinylanisole (−11.44 kcal/mol) and *trans*-phytol (−4.10 kcal/mol) (Table 2). Heptacosane forms two alkyl interactions with Arg 47 and Lys 45 in addition to the formation of multiple hydrophobic interactions and its size that fits the active site. Meanwhile, four H-bonds with Asp 305, Glu 113 and Ser 304, π-π interaction with Trp 303, Phe 46, π-δ interaction with Tyr 55 in addition to π-alkyl interaction with Pro 18 (Figure 7).

### 3.4. In Vitro Assessment of the Anti-Ageing Potential of Premna odorata Leaves Essential Oil

**Figure 8:**
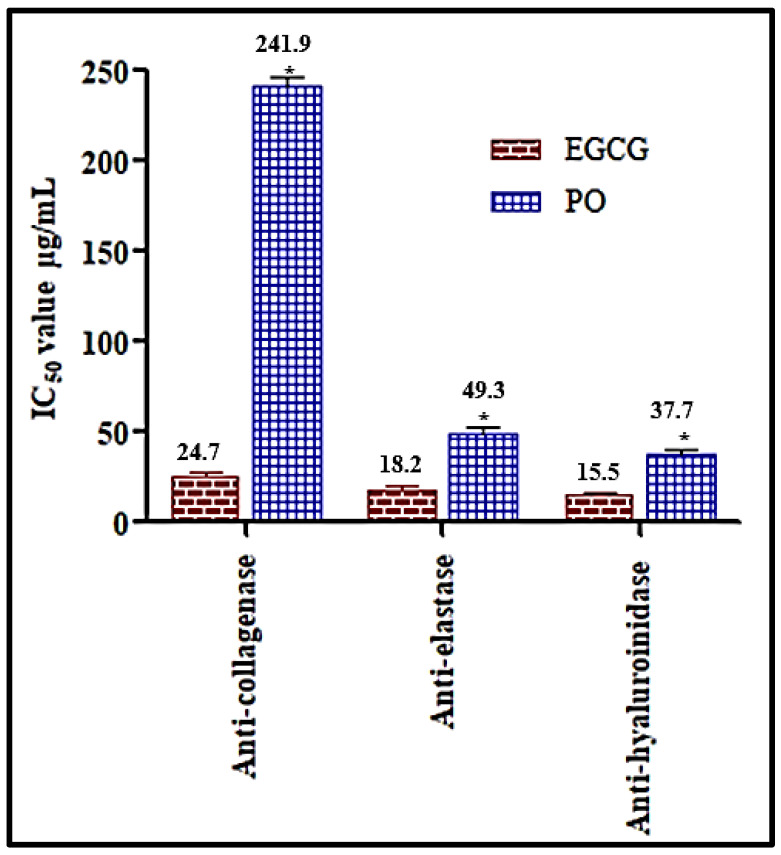
In vitro anti-ageing potential of the essential oil of *Premna odorata* leaves (PO) using anti-collagenase, anti-elastase and anti-hyaluronidase versus EGCG.

Molecular modelling study shows that different compounds existing in the essential oil of *Premna odorata* leaves revealed promising fitting scores so the different samples were mixed in equal volumes (PO) and subjected to in vitro assays namely, anti-collagenase, anti-elastase and anti-hyaluronidase assays. The essential oil shows considerable anti-elastase and anti-hyaluronidase potential as evidenced by their IC_50_ values being 49.3 and 37.7 μg/mL, respectively, approaching that of EGCG, the standard anti-ageing drug, that shows IC_50_ values equal to 18.2 and 15.5 μg/mL for elastase and hyaluronidase, respectively. Meanwhile, the essential oil of *Premna odorata* leaves displayed mild anti-collagenase with IC_50_ value equals to 241.9 μg/mL whereas EGCG shows IC_50_ value equals 24.7 μg/mL (Figure 8). This, in turn, further consolidates the results obtained from molecular modelling and supported the previously reported results that palmitic, oleic and linoleic acid exerted potent anti-ageing potential. In addition to their inhibitory potential to ageing enzymes, and moisture loss prevention, oleic acid showed asignificant antioxidant behaviour [16]. It is noteworthy to highlight that *β*-caryophyllene, the major constituent in the oil, has previously showed a significant anti-ageing potential in *C. elegans* models evidenced by increasing the longevity in its lifespan by more than 22% and was found to be attributed to its antioxidant activity where it reduced the levels of the free radicals level [17]. Thus, the oil exhibited anti-ageing potential may be attributed to the synergistic action of all metabolites as each may act with a different mechanism.

## 4. Conclusions

The essential oil composition of *Premna odorata* leaves obtained from different seasons showed quantitatively and qualitatively metabolic variation as determined employing GC/MS as well as GC/FID techniques. The picture of this metabolic variation was clear when GC data was coupled with PCA as an unsupervised technique of chemometric analysis. Furthermore, molecular modelling on collagenase, elastase and hyaluronidase enzymes active centres shows that different compounds existing in the essential oil of *Premna odorata* leaves shows binding to the active sites with variable degrees that suggested its anti-ageing potential. This was further consolidated by in vitro assays that confirmed the obtained results. Thus, it can be concluded that *Premna odorata* could serve as a promising anti-ageing naturally occurring drug that could be effectively incorporated by pharmaceutical industries in cosmetics combating ageing and skin wrinkling. Further studies are recommended to be done to make comparative studies between the anti-ageing potential of each of the oils sample alone collected in each season relative to the activity of their mixture.

## Figures and Tables

**Table 1:** Essential oil compositions of POS (spring), POM (summer), POA (autumn) and POW (winter) of *Premna odorata* leaves.

Compound		RI		Content [%]				Identification Method
		Cal.	Rep.	POS	POM	POA	POW	
**1.**	*α*-Pinene	927	927	1.95	0.89	6.85	0.15	MS, RI, AU
**2.**	Camphene	944	946	-	-	0.04	-	MS, RI, AU
**3.**	2-Methylnonane	960	962	-	-	tr	-	MS, RI
**4.**	Sabinene	971	971	0.13	0.07	0.36	-	MS, RI
**5.**	*β*-Pinene	975	975	tr	-	0.08	-	MS, RI, AU
**6.**	3-Octenol	979	979	0.87	0.81	0.58	6.52	MS, RI
**7.**	3-Octanone	987	987	tr	0.2	-	-	MS, RI
**8.**	*β*-Myrcene	992	992	0.24	-	0.55	0.14	MS, RI, AU
**9.**	3-Octanol	997	997	0.05	-	-	tr	MS, RI
**10.**	*n*-Decane	1000	1000	0.08	0.23	0.23	0.14	MS, RI
**11.**	*α-*Phellandrene	1005	1005	0.95	0.61	1.57	0.49	MS, RI, AU
**12.**	3-Carene	1011	1011	tr	-	0.05	-	MS, RI
**13.**	*α*-Terpinene	1018	1018	0.47	0.15	0.86	0.25	MS, RI, AU
**14.**	*o*-Cymene	1027	1028	0.57	0.26	2.16	0.99	MS, RI
**15.**	Limonene	1032	1032	8.34	0.82	14	6.95	MS, RI, AU
**16.**	*β*-cis-Ocimene	1050	1051	tr	7.4	0.07	tr	MS, RI
**17.**	*trans-*Decaline	1056	1056	tr	-	0.06	tr	MS, RI
**18.**	*τ-*Terpinene	1061	1061	0.39	0.05	-	0.38	MS, RI
**19.**	1-Octanol	1074	1074	tr	-	-	tr	MS, RI
**20.**	Linalool oxide	1075	1075	tr	-	-	tr	MS, RI
**21.**	*α*-Terpinolene	1091	1088	0.16	0.4	0.13	-	MS, RI
**22.**	*n*-Undecane	1101	1100	0.09	-	-	-	MS, RI
**23.**	*β*-Linalool	1103	1103	0.79	0.08	0.42	1.21	MS, RI, AU
**24.**	Nonanal	1107	1107	0.12	0.61	0.11	0.29	MS, RI
**25.**	*cis-β*-Terpineol	1125	1125	0.06	-	-	-	MS, RI
**26.**	4-Isopropyl-1-methyl-2 cyclohexen-1-ol	1143	1142	tr	0.11	tr	tr	MS, RI
**27.**	Lilac aldehyde B	1146	1148	tr	-	-	-	MS, RI
**28.**	Verbenol	1149	1148	tr	-	-	-	MS, RI
**29.**	*p*-Vinylanisole	1156	1159	0.43	0.24	0.24	1.04	MS, RI
**30.**	*trans*-2-Nonenal	1162	1162	0.08	-	-	-	MS, RI
**31.**	Terpinen-4-ol	1182	1182	0.16	0.28	0.1	0.58	MS, RI
**32.**	Cryptone	1191	1188	0.04	-	-	tr	MS, RI
**33.**	*α*-Terpineol	1195	1195	0.06	0.15	tr	tr	MS, RI
**34.**	Methyl salicylate	1199	1198	0.28	0.1	tr	0.19	MS, RI
**35.**	*n*-Dodecane	1200	1200	-	0.09	0.07	0.18	MS, RI
**36.**	Decanal	1207	1207	-	-	tr	-	MS, RI
**37.**	*trans*-Piperitol	1212	1211	tr	-	-	tr	MS, RI
**38.**	2,4-Nonadien-1-al	1218	1218	tr	-	-	-	MS, RI
**39.**	*β*-Cyclocitral	1227	1223	tr	-	tr	-	MS, RI
**40.**	Hexyl 2-methylbutanoate	1240	1243	tr	-	-	-	MS, RI
**41.**	*p*-Menth-4-en-3-one	1255	1251	tr	-	-	-	MS, RI
**42.**	*trans*-Geraniol	1258	1258	-	-	tr	tr	MS, RI
**43.**	*trans*-2-Decenal	1266	1266	tr	0.11	0.04	0.14	MS, RI
**44.**	Phellandral	1282	1276	-	-	0.08	0.15	MS, RI
**45.**	Bornyl acetate	1293	1293	0.09	0.09	0.08	tr	MS, RI
**46.**	Edulan I, dihydro-	1302	1292	0.16	0.14	0.1	0.49	MS, RI
**47.**	Carvacrol	1307	1307	-	tr	tr	tr	MS, RI
**48.**	*p*-Vinylguaiacol	1320	1320	-	-	0.08	0.19	MS, RI
**49.**	Citronellol acetate	1357	1357	0.22	0.26	-	-	MS, RI
**50.**	Longicyclene	1361	1371	tr	-	-	-	MS, RI
**51.**	*α-*Copaene	1384	1383	3.04	2.21	2.08	2.21	MS, RI
**52.**	*β-*Damascenone	1391	1391	0.14	-	0.23	-	MS, RI
**53.**	*β-*Elemene	1399	1398	1.26	0.91	0.66	0.94	MS, RI
**54.**	Caryophyllene	1415	1415	35.37	26.39	18.84	26.97	MS, RI, AU
**55.**	*τ*-Muurolene	1433	1433	0.16	-	0.17	0.23	MS, RI
**56.**	Sesquichamene	1441	1443	0.09	-	-	-	MS, RI
**57.**	*α-*Himachalene	1444	1438	tr	-	-	-	MS, RI
**58.**	*α-*Humulene	1453	1451	7.29	5.57	4.01	5.75	MS, RI
**59.**	*trans-*Geranylacetone	1458	1458	-	tr	-	-	MS, RI
**60.**	*τ*-Muurolene	1468	1468	0.24	0.24	-	-	MS, RI
**61.**	Germacrene D	1490	1491	10.21	6.35	4.02	5.65	MS, RI
**62.**	*α*-Selinene	1496	1496	0.14	-	-	-	MS, RI
**63.**	*β-*Eudesmene	1498	1495	-	-	0.09	-	MS, RI
**64.**	*α-*Muurolene	1501	1500	0.34	-	0.13	0.4	MS, RI
**65.**	*α-*Farnesene	1512	1508	0.18	0.2	0.14	0.45	MS, RI
**66.**	Eremophilene	1515	1515	0.82	-	0.24	-	MS, RI
**67.**	*τ-*Cadinene	1520	1530	0.33	-	0.17	0.16	MS, RI
**68.**	*β-*Cadinene	1534	1529	7.82	7.12	5.5	7.71	MS, RI
**69.**	Caryophyllene oxide	1537	1537	1.32	2.31	1.46	3.75	MS, RI, AU
**70.**	*α-*Calacorene	1554	1553	-	0.09	0.09	0.13	MS, RI
**71.**	*trans-*Nerolidol	1566	1566	0.04	0.31	-	0.39	MS, RI
**72.**	Germacrene D-4-ol	1570	1570	0.14	0.27	-	-	MS, RI
**73.**	Spathulenol	1589	1589	-	tr	-	-	MS, RI
**74.**	Widdrol	1601	1606	0.06	-	-	-	MS, RI
**75.**	Cubenol	1642	1642	0.08	0.15	0.09	0.24	MS, RI
**76.**	*τ-*Muurolol	1656	1655	0.37	0.41	0.24	0.58	MS, RI
**77.**	*δ-*Cadinol	1659	1655	-	-	0.08	-	MS, RI
**78.**	*α-*Cadinol	1670	1676	0.5	0.72	0.42	1.13	MS, RI
**79.**	Pentadecanal	1720	1721	0.62	-	-	-	MS, RI
**80.**	*trans*-Farnesol	1729	1722	-	0.14	-	0.21	MS, RI
**81.**	Myristic acid	1765	1767	-	0.18	0.38	0.2	MS, RI
**82.**	cis-9-Hexadecenal	1798	1800	tr	-	-	-	MS, RI
**83.**	Hexadecanal	1820	1819	0.08	-	-	-	MS, RI
**84.**	Hexahydrofarnesyl acetone	1849	1848	0.15	0.09	0.05	0.17	MS, RI
**85.**	Palmitic acid	1973	1973	1.03	7.99	5.75	3.06	MS, RI, AU
**86.**	*trans*-Phytol	2127	2122	3.91	3.05	0.95	2	MS, RI
**87.**	Oleic Acid	2156	2152	0.07	0.08	-	8.9	MS, RI, AU
**88.**	Linoleic acid	2161	2157	-	11.92	-	-	MS, RI, AU
**89.**	Linolenic acid	2170	2178	-	-	15.76	-	MS, RI, AU
**90.**	Stearyl acetate	2219	2122	-	0.34	-	-	MS, RI
**91.**	Docosane	2208	2200	0.07	-	0.13	-	MS, RI
**92.**	Tricosane	2305	2300	Tr.	-	0.2	0.27	MS, RI
**93.**	Tetracosane	2398	2400	-	-	0.14	-	MS, RI
**94.**	Pentacosane	2505	2500	0.22	0.21	0.25	0.25	MS, RI
**95.**	Hexacosane	2606	2600	0.08	-	0.09	0.41	MS, RI
**96.**	Heptacosane	2706	2700	1.15	0.87	0.59	-	MS, RI
**97.**	Octacosane	2799	2800	0.09	-	0.09	-	MS, RI
Monoterpene hydrocarbons				13.26	10.65	26.72	9.35	
Oxygenated monoterpene				1.91	1.35	1.1	3.66	
Sesquiterpene hydrocarbons				67.29	49.08	36.14	50.6	
Oxygenated sesquiterpene				3.28	4.4	2.34	6.47	
Others				8.45	26.79	25.65	22.55	
Total identified components				94.19	92.27	91.95	92.63	

**Table 2:** Free binding energies (∆G) of the major identified compounds in collagenase, elastase and hyaluronidase enzymes active centres using molecular docking studies and expressed in kcal/mol employing both pH-based and rule based ionization methods.

Compound	Collagenase		Elastase		Hyaluronidase	
	pH-Based	Rule-Based	pH-Based	Rule-Based	pH-Based	Rule-Based
*α*-Pinene	4.99	4.99	9.63	9.63	13.44	12.22
*α-*Phellandrene	6.85	6.85	6.46	6.46	16.45	16.45
Limonene	14.69	13.92	14.65	16.74	24.90	20.40
*β*-cis-Ocimene	11.85	11.85	17.84	17.84	22.06	22.06
*β*-Linalool	−3.84	0.10	9.34	6.43	12.07	12.40
*p*-Vinylanisole	−20.06	−20.06	−15.56	−15.56	−11.44	−11.44
*β-*Elemen	21.19	21.19	26.71	26.71	33.61	33.61
Caryophyllene	11.74	11.74	11.48	11.48	28.20	28.20
*α-*Humulene	46.58	46.58	49.96	50.07	56.05	56.05
Germacrene D	1.15	1.15	6.24	6.24	11.47	11.47
*β-*Cadinene	27.90	27.90	27.03	27.03	36.47	36.47
Caryophyllene oxide	−7.19	−5.20	−1.27	0.92	4.57	6.13
*α-*Cadinol	7.29	7.29	15.96	15.96	22.23	22.23
Palmitic acid	−78.27	−78.27	−45.77	−45.77	−40.85	−39.19
Oleic Acid	−57.81	−57.81	−31.50	−31.50	−31.59	−31.59
*trans*-Phytol	−28.44	−29.65	−16.62	−15.12	−4.10	−4.70
Linoleic acid	−59.31	−59.31	−31.51	−31.51	−30.48	−30.48
Linolenic acid	−29.85	−29.85	−1.43	−1.43	4.12	4.12
Heptacosane	−63.64	−63.64	−40.48	−40.48	−43.78	−43.78
Epigallocatechin gallate	−59.27	−59.27	−49.35	−49.35	−43.70	−43.70

Positive values indicate unfavourable interaction.
